# Can Smart Home Technologies Help Older Adults Manage Their Chronic Condition? A Systematic Literature Review

**DOI:** 10.3390/ijerph20021205

**Published:** 2023-01-10

**Authors:** Gabriella Facchinetti, Giorgia Petrucci, Beatrice Albanesi, Maria Grazia De Marinis, Michela Piredda

**Affiliations:** 1 Research Unit of Nursing Science, Department of Medicine and Surgery, Campus Bio-Medico di Roma University, Via Alvaro del Portillo, 00128 Rome, Italy; 2 Department of Orthopaedic and Trauma Surgery, Campus Bio-Medico di Roma University, Via Alvaro del Portillo, 00128 Rome, Italy; 3 Department of Public Health and Pediatrics, University of Turin, Via Santena 5 bis, 10126 Turin, Italy; 4 Campus Bio-Medico University Hospital Foundation, Via Alvaro del Portillo, 00128 Rome, Italy

**Keywords:** older people, smart home, home automation, domotic, gerontechnology, ambient assisted living, ambient intelligence

## Abstract

The management of chronic diseases requires personalized healthcare that allows older adults to manage their diseases at home. This systematic review aimed to describe the smart home technologies used in the management of chronic diseases in older people. A systematic literature review was conducted on four databases and was reported following the PRISMA statement. Nineteen articles were included. The intervention technologies were classified into three groups: smart home, characterized by environmental sensors detecting motion, contact, light, temperature, and humidity; external memory aids, characterized by a partnership between mobile apps and smart home-based activity learning; and hybrid technology, with the integration of multiple technologies, such as devices installed at patients’ homes and telemedicine. The health outcomes evaluated are vital signs, medication management, ADL-IADL, mobility, falls, and quality of life. Smart homes show great potential in the management of chronic diseases by favouring the control of exacerbations and increasing patients’ safety by providing support in disease management, including support for cognitively impaired older people. The use of smart homes in the community could bring numerous benefits in terms of continuity of care, allowing the constant monitoring of older people by local and hospital health services.

## 1. Introduction

Increasing life expectancy, advancements in medical science and technologies, and falling birth and mortality rates are causing a rapid rise in the aging population [1]. Aging is associated with dependence on care services, which brings concerns regarding the provision of long-term care services. As people grow older, health needs tend to become more complex, with a general decline in capacity and increased susceptibility to chronic diseases [2].

The management of chronic diseases requires personalized healthcare solutions [3] that allow older adults to manage their diseases daily at home. Indeed, older people prefer to live independently in their homes as long as possible [4]. This preference is shared by policymakers and health providers to avoid the institutionalization of older adults, which would be much more expensive for healthcare systems [2]. This is known as aging in place, which means that, as people grow older, they are able to continue living in their own homes despite changes in health and mobility [5]. Aging in place aims at meeting people’s wishes and giving them the ability, through the provision of appropriate services and assistance, to continue living relatively independently in the community, in their current home or in appropriate housing, depending on their level of autonomy [6].

New technologies, such as the Internet of Things (IoT) and ambient assisted living, have been shown to have great potential to support aging in place [7]. In particular, smart home technologies aim to promote people’s independence and autonomy in their own homes, empowerment, and social inclusion [8].

Smart home technology is defined as an integration of Internet-enabled digital devices with sensors and machine learning in the home [9] that is able to acquire and apply knowledge about the physical environment and its inhabitants in order to improve their experience in that environment [10].

Smart home technology differs from simple home automation because it incorporates the IoT, defined as a network of physical objects such as sensors, processing ability, software and other technologies that connect and exchange data with other devices and systems over the Internet or other communications networks [11]; and Artificial Intelligence (AI) techniques—including Machine Learning (ML)—that is machine intelligence and the branch of computer science that aims to create it. Five basic characteristics of a smart home are automation, recognized as the ability to perform automatic functions; multifunctionality, the ability to perform various activities; adaptability, the capacity to adapt to user needs; interactivity, the ability to interact with or to allow interaction between users; and efficiency, the ability to perform functions conveniently and quickly [12]. Smart homes are equipped with automated systems that control various features of the home, such as lighting, temperature, multimedia, window and door operations, and activity scanning. The sensitivity of this intelligent environment allows it to respond to modern human and social needs [13]. Smart homes can also respond to health needs by monitoring physiological parameters (pulse, oxygen saturation, blood pressure); functionality (general activities, motion, meal intake); safety and security (automatic lighting, trip and fall reduction, hazard detection, intruder detection); social interaction (phone calls, video-mediated communication, virtual participation in groups); and cognitive/sensory assistance (medication reminder, lost key locator) [14]. With these functionalities, smart home technology could be an effective way to manage chronic diseases at home, by promoting patient engagement and care delivery, if necessary.

Despite the continuous development of technology to promote aging in place, and the fact that several reviews were recently published about the use of the smart home in the everyday lives of older people [15,16,17], and also providing a guide to current sensor technology for unobtrusive in-home monitoring [18], the focus on chronic diseases seems sparse. Indeed, only the systematic review by Liu and colleagues [19] focused on chronic diseases, including studies related only to tele-monitoring and tele-exercise. Few interventions focusing on smart home technologies intended as an integration of Internet-enabled digital devices with sensors and machine learning in the home [9] were reviewed.

Therefore, an integration of the previous systematic review on the impact of smart homes in the management of chronic diseases in older people living at home is warranted.

The present systematic review aimed to describe the smart home technologies used in the management of chronic diseases in older people. The specific research questions were the following:

(1) What are the characteristics and aims of smart home technologies?

(2) What type of health outcomes have been reported?

## 2. Materials and Methods

This systematic review was reported in accordance with the PRISMA statement [20]. The protocol was prospectively registered on PROSPERO (registration number CRD42020137480).

### 2.1. Search Strategy

A comprehensive literature search was performed in February 2022 on the following databases: PubMed, Medline, CINAHL (Cumulative Index to Nursing and Allied Health Literature), and IEEE Xplore (Institute of Electrical and Electronics Engineers Xplore). No language and date restrictions were set. The search strategy was devised by using both a thesaurus and free terms for the following keywords: smart home, home automation, domotic, ambient intelligence, gerontechnology, ambient assisted living, sensor motion detection, in-home monitoring, aged, elderly, geriatric, gerontology, older people, senior, and chronic disease. The search strategy was checked by three reviewers (GF, GP, and BA). Further details about the search strategies used are provided in File S1.

### 2.2. Study Selection and Data Collection

The records retrieved were screened against the following inclusion and exclusion criteria to select quantitative studies focused on smart home technologies—identified by the five basic characteristics established by Lee & Kim [12]—used to monitor older adults (≥65 years) living in their homes and diagnosed with at least one chronic disease. Papers were excluded if they focused on technical aids, such as telehealth devices or digital services, without smart technology. No restrictions on study design and health-related outcomes were set.

Record screening was independently conducted by two reviewers (GF and GP). A first-round screening of titles and abstracts, based on the inclusion criteria, was followed by full-text selection. To maximize the search sensitivity, a snowball method was used [21], and the reference lists of the full texts included were screened. Conflicts regarding study inclusion were solved by discussions and a mutual agreement was achieved between reviewers. The data extracted from the full texts selected (including first author, publication year, country, sample size, patient disease, type of study, type of technology, characteristics of the technologies, and outcomes) were independently extracted by two authors (GP and GF) and checked by a third author (BA).

### 2.3. Quality Assessment

The quality of the studies included was evaluated through the Quality Assessment Tool for Observational Cohort and Cross-Sectional Studies, Case Studies, and Randomized Controlled Trials developed by the National Heart, Lung, and Blood Institute (https://www.nhlbi.nih.gov/health_topics/study_quality_assessment_tools, accessed on 2 march 2022). This tool considered several quality criteria (Table 1 and File S2). Each criterion was evaluated in response to the tool’s questions, assigning Cannot Determine (CD), No (N), Not Reported (NR), or Yes (Y). Quasi-experimental studies were assessed with the Joanna Briggs Assessment Tool for Quasi-experimental studies [22]. Based on this evaluation, the studies were classified into three levels of quality rating: good (with only 1 CD, N or NR), fair (with 2 CD, N or NR), and poor (with >2 CD, N or NR).

### 2.4. Data Analysis

A meta-analysis could not be performed due to the limited data available. A narrative synthesis was carried out describing the study, patient, and intervention characteristics. Descriptive analyses of categorical data were reported as percentages and frequencies.

## 3. Results

**Figure 1:**
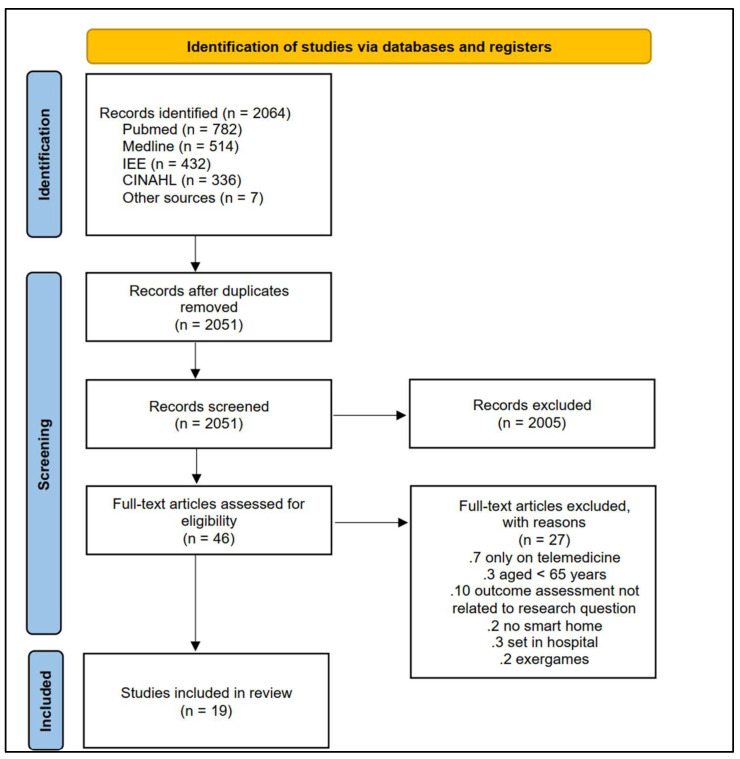
PRISMA flow chart of search strategy.

The article selection process is illustrated in Figure 1. A total of 2064 articles were retrieved. After duplicate removal, the titles and abstracts were screened, and the full texts of potentially eligible studies were retrieved. Nineteen articles were included and evaluated for methodological quality.

### 3.1. Study Types and Patient Characteristics

The designs of the 19 included studies were as follow: case studies/case series studies (n = 6; 31.57%), quasi-experimental studies (n = 4; 21.05%), iterative studies (n = 1; 5.26%), observational studies (n = 5; 26.31%), and RCTs (n = 3; 15.78%).

A total sample of 1383 older patients were reviewed. The patients were affected by the following chronic diseases: heart disease (n = 679; 49.09%), multi-chronic diseases such as diabetes, asthma, and hypertension (n = 732; 52.92%), and neurodegenerative diseases such as dementia (n = 271; 19.59%). All the studies were published in English in peer-reviewed journals from 2004 to 2020 and were mainly conducted in the USA (n = 7; 37%) (Table 2).

### 3.2. Quality Assessment

No quality assessment was performed for the one iterative study because of the lack of a specific evaluation tool [10,11,12,13,14,15,16,17,18,19,20,21,22,23,24,25,26,27,28,29,30,31,32,33,34,35,36,37,38,39,40]. The remaining 18 studies yielded the following judgements of quality level: good (n = 7, 37%), poor (n = 7, 37%), and fair (n = 4, 21%) (Table 1).

### 3.3. Interventions Characteristics and Aims

All studies detailed the smart home technologies utilized as described below, but only three studies [26,27,28,29,30,31,32,33,34,35,36,37,38,39,40] also described the models/tools used by AI or machine learning (ML) and the IoT. The first one utilized eight different ML models, each of which learns a mapping between a single activity and its corresponding direct observation score. The scoring output of the algorithm was a sum of the eight individual activity scores generated by the eight different learning models. The second one used a combination of AI tools such as Hidden Markov Models; Naive Bayes Classifier; Gaussian Mixture Models. The last used AI tools for behavior feature extraction and ML models for behavior prediction (details in File S3).

Different types of technologies supporting older people’s life were classified into three groups with the following characteristics: (1) Smart Homes (n = 13; 68.42%), characterized by environmental sensors detecting motion, contact, light, temperature, and humidity (including domotic technology); (2) External Memory Aids (n = 1; 5.26%), characterized by a partnership between mobile apps and smart home-based activity learning; and (3) Hybrid Technology (n = 5; 26.31%), characterized by the integration of multiple technologies, such as devices installed at patients’ homes and telemedicine.

### 3.4. Smart Homes

Smart homes use environmental sensors, such as infrared motion, contact, light, temperature, and humidity, to enable the unobtrusive monitoring of residents’ behaviour. Each type of sensor is used in different ways. Motion sensors usually refer to a Passive InfraRed (PIR) sensor, made with pyroelectric materials sensitive to infrared light emitted by the body, that detects the presence of residents in the rooms [7] and when and where they are moving. Some motion sensors detect any movement occurring in an entire room, while others only detect motion occurring near the sensor and within its direct line-of-sight [27]. PIR sensors can provide information on the resident’s location, time spent in a single room, frequency of toilet usage, and sleeping [27]. These detectors are frequently used for monitoring the Activity of Daily Living (ADL) [37]. Contact sensors detect the manipulation of an object using a magnetic switch to capture when two magnets are in proximity to each other, allowing current to flow. When the magnets are close, the alarm is not activated, but when the magnets are distant, the alarm is activated. In this way, the sensor can provide, for example, information about whether a door is open or closed [7]. The door usage behaviour patterns bring information about entering and exiting the home that could be related to residents’ social behaviours [27]. Light sensors located throughout the home provide information on the ambient light level in each room during the day, providing indirect data regarding the resident’s activity levels during the day or night. For example, light sensors placed inside the refrigerator provide information about food-related routines [27]. Lastly, combining sensors that detect temperature, humidity, and motion could provide information on activities involving the use of the kitchen stove and bathroom showers [27].

### 3.5. External Memory Aids

This technology integrates smart homes and mobile devices. In particular, it refers to digital-memory notebook mobile applications using smart homes data and activity recognition techniques. The aim of these applications is to support patients in managing their daily tasks [10], helping them complete their daily activities by providing reminders, and indicating the activities completed and those still to be completed [10]. To achieve its goal, this technology uses the Digital Memory Notebook (DMN) mechanism, which automatically checks off completed tasks using smart home data to reassure individuals about tasks they have completed and predicts when tasks should be completed. To determine the latter, it utilizes information to prompt users to initiate activities at the times they normally occur, without the user programming them [10].

### 3.6. Hybrid Technology 

We classified as hybrid technologies studies that describe solutions linking several devices at home with medical staff through a service of telemedicine. They differ from the telemedicine-only service because they combine smart home sensors with units or monitors provided by devices such as a blood pressure cuff, oximeter, glucometer, and thermometer. Through these devices, several vital parameters of older adults are detected and sent to medical staff by the telemedicine service.

The studies by Celler et al. [24], Soran et al. [36], and Wakefield 2014 [38] concern home-based management systems to monitor and detect early signs and symptoms. Celler et al. [24] describe devices with friendly user interfaces able to detect a range of vital signs, allow patient video-conferencing and messaging capability, fill out Clinical Questionnaires specific to patient condition, and deliver Educational/training material.

Goldberg et al. [28] describe a system called AlertNet that includes an electronic scale placed in patients’ homes and an individualized symptom response system linked via a standard phone line using a toll-free telephone number to a computerized database monitored by trained cardiac nurses. Patients were instructed to weigh themselves and respond to questions about symptoms related to their chronic disease twice a day. Any change in weight and symptoms is monitored by the nurse who, in case of need, contacts the patient and the physician.

A complex system installed in patients’ homes, inspired by the Wagner model, was cited by Kuo et al. [31]. This system, tracing and monitoring health status 24/7 in real-time, measures several physiological parameters. Moreover, an emergency call system is embedded into a health pad to help patients in emergencies. A link with a system for drug prescription delivery and social welfare services applications, allowing connection to the hospital information system and the social welfare databases, is also integrated. Healthcare professionals will take charge of the application process, such as document preparation and transfers to government authorities.

### 3.7. Health Outcomes and Vital Signs

Several health outcomes were evaluated in the studies included, such as vital signs (n = 3, 15.78%), medication management (n = 2, 10.52%), ADL (Activity of Daily Living)/IADL (Instrumental Activity of Daily Living) (n = 10, 52.63%), mobility and falls (n = 4, 21.05%), and quality of life (n = 2, 10.52%).

Devices installed in patients’ homes detected vital signs, such as blood glucose, peripheral oxygen saturation, blood pressure, and body temperature. These were used to remotely monitor the patient’s health status by a nurse who could intervene in case of need. One study showed that smart home technology could significantly reduce daily abnormal Blood Pressure (BP) by encouraging proper daily BP measurement [31]. Celler et al. [24] demonstrated significantly better glycaemic control in diabetic patients who transmitted blood glucose and blood pressure data to a telehealth nurse.

### 3.8. Medication Management 

One study described a system utilizing unobtrusive online, mobile, wearable devices and sensors for feedback and intelligent analysis in an ambient assisted living environment, embedded in the kitchen, bedroom, and an unspecified environment, [32] that supports patients with cognitive impairment to manage their medication through medication management-feedback from User Interface-messages from the clinician.

A Smart Home for Elders (SHfE) technology [39] recorded the time when patients took their medicine during the day by activating when the medicine box opened thanks to switch sensors set up on a medical kit that detected the opening and closing of the box. Furthermore, SHfE advised participants when they did not take their medicine on time.

### 3.9. IADL and ADL

Irregularities in ADL performance, as well as dysfunctions in daily routine, can be recognized and quantified using unobtrusive sensor-based recognition systems and activity map-based visualization techniques [37]. A multi-sensor system can be used 24 h a day for the assessment of older people’s behaviours, allowing staff to monitor them permanently. It can also predict when people get up after in-bed restlessness and make a first dangerous-event prediction by analysing correlations and relationships between in-bed restlessness and getting up [25]. Furthermore, with data collected from bathroom sensors, it is possible to determine when someone is showering or using the toilet and for how long [27]. Another study reveals that smart home technologies can detect over time improvements in moving intensity, ADLs, cognitive function, and behavioural aspects such as night-time sleep for all participants [32].

### 3.10. Mobility and Falls

Several chronic diseases can induce the degeneration of patients’ self-perception in space. One study continually analysed patients’ posture and motion, as their ability to move correctly and safely in the environment allows caregivers to check the patient’s locomotion capabilities and intervene at the right time in case of necessity (i.e., when the patient falls down) [23]. Another study predicted mobility, cognitive, and mood-related symptoms from unobtrusively collected in-home behaviour data, revealing that mobility was related to sleep disorders [40] and that walking times of subjects with mild cognitive impairment were longer in the evening, as compared with the healthy controls [29].

### 3.11. Quality of Life

In a randomized controlled trial with patients affected by heart failure (HF), quality of life at three months was better in telehome-monitored patients than in usual-care patients on five out of the eight SF-12 subscales [28]. The magnitude of the absolute differences in quality of life between intervention and control groups ranged from 4% to 15%. The only subscale on which telehome-monitored patients had significantly better improvement in quality of life at all three points in time (one month, three months, and one year after discharge) was the vitality subscale, which reflects the subject’s energy level and fatigue. Patients with HF in both randomized groups demonstrated significant improvements in quality of life over time in all the SF-12 subscales. General health was better in telehome than in usual-care patients at one and three months post-discharge. At one year, quality of life was higher in telehome than in usual care patients [28]. Another study showed that a system for continuous and objective remote monitoring was correlated with less television usage in participants’ daily lives, which is a hallmark of aging well and an improvement in their quality of life [32].

## 4. Discussion

Most studies in this systematic review had a low level of evidence (case studies and quasi-experimental studies) but a good quality level. The study population was affected by heart and multi-chronic diseases, which require constant monitoring due to the potential and frequent exacerbation of symptoms [41]. This systematic review aimed to describe the smart home technologies used in the management of chronic diseases in older people, and at the beginning of the paper proposed to answer the following questions:

(1) What are the characteristics and aims of smart home technologies?

(2) What type of health outcomes have been reported?

### 4.1. What Are the Characteristics and Aims of Smart Home Technologies?

Most of the current studies have shown encouraging results, confirming that a combination of AI and IoT can help older people live easier and better lives [17]. Increasingly, AI-IoT-enhanced interventions are being developed to support the health and capacity of older people, with the aim of expanding the reach of care and its efficiency. These technologies have the potential to improve the sustainability of the workforce (e.g., by serving as additional support for healthcare professionals), enable older people to age in place [27], increase the efficiency of information systems and analytics of data on the progression of chronic diseases of the elderly [42].

The types of technology found in this review have been assembled into three groups. The first group includes smart home technology with different capabilities for monitoring and managing the health of older adults. Smart homes have a high impact on older people’s daily living [15] and activities in terms of promoting physical activity [43] and a sense of security [44] that are fundamental to reducing hospitalizations and admissions to residential structures [43,45].

Smart homes allow older people, even with cognitive impairment, to stay at home for as long as possible in complete safety. Numerous studies indicate that older adults prefer to age in their own homes rather than move to assisted residences [4]. In addition, the sense of safety is extended to family caregivers who can monitor their loved one’s activities at home. The implications related to the privacy of the person could discourage the use of this technology. A qualitative study assessed older adults’ perceptions and expectations about smart home technologies, including perceived advantages and disadvantages and the degree of willingness to adopt such technologies in their homes. It revealed that participants had a positive attitude toward smart home technologies in general. In fact, they understood the potential of this technology to play a reactive role (e.g., detecting emergencies and falls) rather than a proactive one (monitoring the environment and detecting or predicting problems or concerns). Furthermore, they believed that none of the technologies presented to them would interfere with daily activities and showed a desire to have this technology in their home, too [46].

Various barriers to the adoption of smart home technology have been identified. These include usability, accessibility, reliability, trust, stigma, control, privacy, lack of human responsiveness, the burden on others, lack of perceived need, and convenience [18]. Furthermore, in a survey of 661 older people with chronic diseases, more than half of them did not find the smart home beneficial and were not willing to buy this technology [47]. Therefore, the question when building technologies for the elderly should be: are we developing the technologies that older people want? An interesting qualitative study by Ghorayeb et al. [18] attempted to answer this question. This study found that older people preferred unobtrusive, ‘self-taught’ technology and they must be able to know how, where, and what kind of information is transmitted. Older people need to see the benefit of the technology, be able to customize it, and have control over it. Furthermore, any new technology should include a discussion with family members and carers and promote communication, exercise, and social interaction. Indeed, smart homes should not replace social interactions with family members, caregivers, and nurses, but rather, they should facilitate new social and community relationships and events.

Technology such as External Memory Aids, with data derived from smart homes, demonstrates its utility in improving traditional interventions used to help older adults with memory impairments. Due to their capacity for calendaring and programming, this kind of technology seems to act positively in supporting medication management. A study shows that technology with digital prompts and reminder messages can be very helpful in managing medication in patients with chronic diseases [48]. Moreover, these types of technology can act to improve space-time orientation and neuropsychological status by reminding patients of activities, days, and dates [32]. The study conducted by Lazarou et al. [32] showed that the use of such technology improves patients’ cognitive functionality. Although few studies in this review addressed cognitive functionality, it seems that a smart home achieves positive results. In particular, sleep monitoring allows the management or prevention of delirium or states of nocturnal agitation [26,49].

Hybrid technology is useful for detecting vital signs in home environments and to send them to medical staff. In fact, the patients who were included in this technology group showed an improvement in the control of vital signs [50]. This can dramatically improve the time efficiency of emergency delivery for acute patients [30].

### 4.2. What Type of Health Outcomes Have Been Reported?

Several of the health outcomes analysed were managed with the use of smart home. One that consistently emerges is the ability of a smart home to help in the detection of patients’ ADL, which can be useful in assisted living and structuring behavioural and lifestyle profiles [19,51]. A smart home, through its sensors and its map-based visualization techniques, can provide a complete image of older people’s activities, adapting the home to their ‘life rhythms’ [48]. Furthermore, the detection of ADL is crucial due to the chronicity and complications that characterize older patients, in order to intervene and improve health behaviours. Thanks to monitoring of patient mobility, posture, and sleep disorders, it becomes possible to implement specific interventions to prevent or manage accidental falls, by creating an external warning system [52]. Moreover, given the possibility of the remote evaluation of smart homes, it could be possible to intervene not only on patients, but also on caregivers, by promoting their participation and/or reducing their burden [53,54]. According to the study by Majumder et al. [55], these are very important elements that can determine an overall perspective on interventions in the family unit.

Another outcome analysed by this review was quality of life. Smart home technologies and hybrid technologies can improve patients’ quality of life and sense of well-being [56], confirming similar effectiveness in patients living in residential care facilities [57,58]. This is also in line with a previous review showing their positive effect on the overall quality of life by helping patients save their energy, perform essential activities more easily, and avoid fatigue [12]. In fact, smart homes influence patients’ healthy aging due to continuous and objective remote monitoring. Moreover, this review provides evidence regarding reduced television usage in participants’ daily lives, which is a hallmark of aging well and improvement in quality of life [32].

### 4.3. Recommendation for Further Research

There are several gaps in our knowledge around smart home technologies in older people that follow from our findings and would benefit from further research. In fact, despite a large number of studies in the literature on smart home technologies, as shown by this review, there are few specific studies with a good level of evidence on older people who live at home with one or more chronic diseases. This indicates the need for more experimental studies with higher levels of evidence in this population in order to arrive at proven efficacy results. In addition, the considerable heterogeneity of study designs, sample sizes, the role of technology, outcome measurement, and reporting make it more difficult to draw conclusions about the effectiveness of smart home technologies, and this should be considered in future evidence-based recommendations.

Research outcomes from smart home interventions should be standardized so that results are more easily comparable. Regarding reporting, future research should choose and follow a single reporting system to reduce reporting bias. Finally, it is essential that the solutions included in future studies are the most appropriate for the needs of the elderly, always recognizing personalization of care as the cornerstone around which these types of technology should rotate, otherwise they risk not being effective, acceptable solutions for aging in place.

### 4.4. Limitations

This review has some limitations. First, despite the intention of a comprehensive literature search, some keywords may have been omitted. We only included studies that had been published through the peer-review process. Therefore, grey literature, such as unpublished documents and reports, were not identified. Moreover, while studies on smart home technologies are more and more numerous, the lack of alignment in the data collection instruments led to high data heterogeneity, which makes it difficult to carry out any type of quantitative analysis.

## 5. Conclusions

This article adds knowledge about smart home technologies used in the homes of older people suffering from chronic diseases to monitor and manage these pathologies, providing a clear classification of what types of smart homes are currently used and what their goals are. It also describes which health outcomes these technologies can monitor or prevent.

Smart home technologies show great potential in the management of chronic diseases at home, not only favouring the control of exacerbations of chronic diseases but also providing safety to patients by supporting them in autonomous disease management. Furthermore, smart homes improve patients’ quality of life and, thus, pursue the main goal of health care in chronic conditions. The use of smart homes as a support tool for the management of chronicity within the community could bring numerous benefits in terms of continuity of care, allowing constant monitoring of older adults by the local and hospital health services.

We hope that this contribution will attract attention and commitment from academia and industry to improve research and investment in smart home technologies and thus enable these technologies to be applied on a large scale to help resolve the challenges of the aging population.

## Figures and Tables

**Table 1:** Summary of quality assessment.

	Good Quality	Fair Quality	Poor Quality
Cavallo et al., 2015 [23]		X	
Celler et al., 2014 [24]	X		
Chan et al., 2005 [25]			X
Dawadi et al., 2016 [26]			X
Fritz et al., 2019 [27]	X		
Goldberg et al., 2003 [28]			X
Hayes et al., 2008 [29]	X		
Jekel et al., 2016 [30]	X		
Kuo et al., 2012 [31]	X		
Lanzarou et al., 2016 [32]	X		
Lanzarou et al., 2019 [33]			X
Rawtaer et al., 2020 [34]	X		
Sacco et al., 2012 [35]		X	
Soran et al., 2010 [36]			X
Urweyler et al., 2017 [37]			X
Wakefield et al., 2014 [38]			X
Yu et al., 2019 [39]		X	

**Table 2:** Characteristics of the included studies (n = 19).

Article Year Country	Sample (n)	Disease	Type of Study	Characteristics of Technology	Aim	Smart Home Functionality	Health Outcomes
Alberdi et al., 2018 [40] Spain	29	Alzheimer disease	Iterative study	Environmental Sensor	To assess the possibility of detecting changes in psychological, cognitive, and behavioural symptoms of AD by making use of unobtrusively collected smart home behaviour data and machine learning techniques	Motion sensors	Sleep disorders, ADL, cognitive abilities, mobility
Cavallo et al., 2015 [23] Italy	14	Alzheimer disease	Case study	Environmental Sensor	To demonstrate the technical effectiveness and acceptability of an innovative domiciliary smart sensor system for providing domiciliary assistance to patients with AD which has been developed with an ambient assisted living approach	Smart sensor system	Exit/entrance monitoring and alerting, multimedia cognitive stimulation, supporting in taking drugs, control of gas and water electron valve, support in using phone
Celler et al., 2014 [24] Australia	375	COPD, Cardiovascular Disease, Diabetes, Asthma	Before After Control Intervention	Integration between Telemedicine and the use of devices	To demonstrate how telehealth services for chronic disease management in the community can be deployed nationally and to develop advanced modelling and data analytics tools to risk stratify patients daily to automatically identify exacerbations of their chronic conditions	Telemedcare monitoring unit	Blood sugar, SpO2, mortality, hospitalization,
Chan et al., 2004 [25] France	4	Dementia and Alzheimer Disease	Case study	Environmental Sensor	To show the detailed results obtained by the system for the differed assessment of night-time activities and the computation of correlation coefficients between data for in-bed rest-lessness and getting up, going out or visiting the toilet in individuals followed for several months	Ten infrared sensors on the ceiling, connected to a computer by means of a communication network in its wire version	Getting up, going out, going to bed, visiting the toilet, in bed restlessness
Dahamen et al., 2018 [10] USA	17	Dementia, memory difficulties	Iterative study	Exergames and Digital Interfaces	To introduce a real-time automated intervention that partners mobile apps with smart home-based activity learning using two primary mechanisms	Digital memory notebook mobile application composed of four main components: home, calendar, profile, and Notes.	ADL (Eat, work cook)
Dawadi et al., 2016 [26] USA	67	Dementia	Observational study	Environmental Sensor	(1) To provide automated task quality scoring from sensor data using machine learning techniques and (2) to automate cognitive health assessment by using machine learning algorithms to classify individuals as cognitively healthy, MCI, or dementia based on the collected sensor data	Motion sensors on the ceiling, door sensors on cabinets and doors, and item sensors	IADL
Fritz et al., 2019 [27] USA	4	Parkinson’s disease	Case series study	Environmental Sensor	To offer practical guidance to nurse investigators interested in multi-disciplinary research that includes assisting in the development of artificial intelligence algorithms for “smart” health management and aging-in-place	Five sensor types: infrared motion, contact, light, temperature, and humidity. The sensors are placed on the ceiling, walls, and doors.	Falls, medications, vital signs, timed up and go test
Goldberg et al., 2003 [28] USA	280	Heart failure with a left ventricular ejection fraction < or =35%	RCT	Integration between Telemedicine and the use of devices	To determine whether daily reporting of weight and symptoms in patients with advanced heart failure reduce rehospitalization and mortality rates despite aggressive guideline-driven heart failure care	AlertNet: a program with the DayLink monitor and an electronic scale	Hospital readmission rate, mortality, emergency room visitation rate, and quality of life
Hayes et al., 2008 [29] USA	14	Alzheimer disease	Quasi-experimental	Environmental sensors	To evaluate the use of continuous, long-term, and unobtrusive in-home monitoring to assess neurological function in healthy and cognitively impaired elders	Motion sensor and contact sensor	Walking speed, mobility
Jekel et al., 2016 [30] Germany	21	Dementia	Pilot study (quasi experimental)	Environmental sensors	To investigate the potential of a smart home environment for the assessment of IADL in MCI	Sensors and video cameras	IADL
Kuo et al., 2012 [31] Taiwan	84	Stroke	Case series study	Integration between Telemedicine and the use of devices	To present an IT-mediated health- care model as an extension of ordinary chronic care	A machine for measuring several physiological parameters (blood pressure, heart rate, blood sugar, and body temperature) is set up in each patient’s home.	Vital signs (Blood pressure, heart rate, blood sugar, body temperature)
Lazarou et al., 2016 [32] Grecee	4	Dementia, mild cognitive impairment	Case study	Environmental sensors	To propose a system for continuous and objective remote monitoring of problematic daily living activity areas and design personalized interventions based on system feedback and clinical observations for improving cognitive function and health-related quality of life	Wearable, sleep, object motion, presence, and utility usage sensor	ADL, cognitive functions, daily functionality
Lanzarou et al., 2019 [33] Grecee	18	Cognitive impairment and Alzheimer’s disease	Observational study	Environmental sensors	(1) To investigate whether the long-term use of sensor-based remote monitoring systems at home can be accepted and sustained (2) To validate the beneficial impact of its long-term use, taking into account the tailored system-driven interventions, among different groups of people with MCI and AD (3) To pilot, maintain, and evaluate the long-term effects (up to a year) of a personalized sensor-based system to support non-pharmacological interventions for people with cognitive impairment, both in preclinical and advanced stages	The Ambient and Wearable Sensors (ambient depth cameras, Plug sensors, tags, presence IRmotion sensor, sleep sensor	Cognitive Functions (memory, attention, etc.) sleep duration and behaviour, physical activity and ADL
Rawtaer et al., 2020 [34] Singapore	49	Mild Cognitive Impairment	Cross-sectional	Environmental sensors	To establish the feasibility and acceptability of utilizing sensors in the homes of senior citizens to detect changes in behaviours unobtrusively	PIR, bed sensor, a sensor-equipped medication box	Time spent away from home, television use, sleep duration
Sacco et al., 2012 [35] France	35	Alzheimer’s disease and mild cognitive impairment	Observational study	Video monitoring system	To assess IADL in AD and in MCI through the video monitoring system	Imaging and video processing enables the patients’ performances and actions in real-time and real-life situations to be captured and accurately evaluated	ADL
Soran et al., 2010 [36] USA	315	Heart failure	RCT	Integration between Telemedicine and the use of devices	To determine if a heart failure disease management program using a computer-based telephonic system for home monitoring, in addition to the targeted and consistent physician/patient education, coupled with assiduous efforts to use optimal medical therapy	Day link monitor: a home-based disease management program to monitor and detect early signs and symptoms of heart failure using telecommunication equipment. The system includes an electronic scale and an individualized symptoms response system linked via a standard phone line to a computerized database staffed by trained nurses	Hospital readmission
Urwyler et al., 2017 [37] Switzerland	20	Dementia	Observational study	Environmental sensors	(1) To investigate the extent of difference in ADL (both basic ADL and IADL) patterns between the healthy controls and dementia patients and to investigate if the difference in ADL can be used to classify the subjects into the two groups (2) To investigate the influence of the measurement duration on the classification performance	Wireless-unobtrusive sensors	ADL
Wakefield et al.,2014 [38] USA	53	Diabetes and Hypertension	RCT	Telemonitoring system + in-home devices	To evaluate the effectiveness of short-term targeted use of remote data transmission on treatment outcomes in patients with diabetes who had either out-of-range haemoglobin A1c (A1c) and/or blood pressure measurements	Electronic monitors and data were transferred to a secure Web site	Blood pressure and glucose blood
Yu et al., 2019 [39] China	1	Chronic diseases	Case study	Environmental sensors	To describe the development of a smart home for elders that uses unobtrusive sensor technology to assess older adults’ daily activities and assist their healthcare services	Wireless sensor networks that value humidity, temperature, electricity usage, closure of doors and windows	ADL

Abbreviations: MCI: mild cognitive impairment; AD: Alzheimer disease; ADL: Activity Daily Living; IADL: Instrumental Activity Daily Living; PIR: Passive InfraRed.

## Data Availability

The labelled dataset used to support the findings of this study is available from the corresponding author upon request.

## Supplementary Materials

The following supporting information can be downloaded at: https://www.mdpi.com/article/10.3390/ijerph20021205/s1, File S1: Search Strategy, File S2: Quality Assessment of included studies, File S3: Description of IoT/AI/ML used in included studies.
