# Supplementary material for: Can Smart Home Technologies Help Older Adults Manage Their Chronic Condition? A Systematic Literature Review
Source: Int J Environ Res Public Health. 2023 Jan 10;20(2):1205. doi: 10.3390/ijerph20021205 (PMC9859495; doi:10.3390/ijerph20021205)
Supplement: Supplementary file 1 [file ijerph-20-01205-s001.zip › File S1_search strategy.pdf]

Appendix 1: **List of search terms used for systematic review**

Search terms for PUBMED: to February 2022

1. smart home
2. home automation
3. domotic
4. ambient intelligence
5. gerontechnology
6. ambient assisted living
7. sensor motion detection
8. in-home monitoring
9. 1 or 2 or 3 or 4 or 5 or 6 or 7 or 8
10. aged[Mesh]
11. aged
12. elderly
13. geriatric
14. gerontology
15. older people
16. senior
17. 10 or 12 or 13 or 14 or 15 or 16
18. 9 and 17

Search terms for MEDLINE: to February 2022

1. smart home
2. home automation
3. domotic
4. ambient intelligence
5. gerontechnology
6. ambient assisted living
7. sensor motion detection
8. in-home monitoring
9. 1 or 2 or 3 or 4 or 5 or 6 or 7 or 8
10. aged[Mesh]

11. aged
12. elderly
13. geriatric
14. gerontology
15. older people
16. senior
17. 10 or 12 or 13 or 14 or 15 or 16
18. 9 and 17

<u>Search terms for IEEE: to February 2022</u>

1. smart home
2. domotic
3. 1 or 2
4. aged
5. older people
6. elderly
7. gerontechnology
8. 4 or 5 or 6 or 7
9. 3 and 7

<u>Search terms for CINHAL: to February 2022</u>

1. smart home
2. home automation
3. domotic
4. ambient intelligence
5. gerontechnology
6. ambient assisted living
7. sensor motion detection
8. in-home monitoring
9. 1 or 2 or 3 or 4 or 5 or 6 or 7 or 8
10. Aged[Mesh]
11. aged

12. elderly
13. geriatric
14. gerontology
15. older people
16. senior
17. 10 or 12 or 13 or 14 or 15 or 16
18. 9 and 17
