# Supplementary material for: Can Smart Home Technologies Help Older Adults Manage Their Chronic Condition? A Systematic Literature Review
Source: Int J Environ Res Public Health. 2023 Jan 10;20(2):1205. doi: 10.3390/ijerph20021205 (PMC9859495; doi:10.3390/ijerph20021205)
Supplement: Supplementary file 1 [file ijerph-20-01205-s001.zip › File S2_quality evaluation.pdf]

Table 2: Quality assessment of included studies

**Observational study**	Urwyler et al., 2017	Dawadi et al., 2013
1. Was the research question or objective in this paper clearly stated?	YES	YES
2. Was the study population clearly specified and defined?	YES	YES
3. Was the participation rate of eligible persons at least 50%?	YES	YES
4. Were all the subjects selected or recruited from the same or similar populations (including the same time period)? Were inclusion and exclusion criteria for being in the study prespecified and applied uniformly to all participants?	YES	YES
5. Was a sample size justification, power description, or variance and effect estimates provided?	NO	YES
6. For the analyses in this paper, were the exposure(s) of interest measured prior to the outcome(s) being measured?	YES	YES
7. Was the timeframe sufficient so that one could reasonably expect to see an association between exposure and outcome if it existed?	YES	YES
8. For exposures that can vary in amount or level, did the study examine different levels of the exposure as related to the outcome (e.g., categories of exposure, or exposure measured as continuous variable)?	NA	NA
9. Were the exposure measures (independent variables) clearly defined, valid, reliable, and implemented consistently across all study participants?	YES	YES
10. Was the exposure(s) assessed more than once over time?	YES	YES
11. Were the outcome measures (dependent variables) clearly defined, valid, reliable, and implemented consistently across all study participants?	YES	YES
12. Were the outcome assessors blinded to the exposure status of participants?	NA	NO
13. Was loss to follow-up after baseline 20% or less?	YES	NA
14. Were key potential confounding variables measured and adjusted statistically for their impact on the relationship between exposure(s) and outcome(s)?	NA	NR
**TOTAL SCORE**	**POOR**	**POOR**

Case Study / Case Series Study	Cavallo et al., 2015	Chan et al., 2004	Lazarou et al., 2016	Kuo et al., 2012	Fritz et al., 2018	Yu et al., 2019
1. Was the study question or objective clearly stated?	YES	YES	YES	YES	YES	YES
2. Was the study population clearly and fully described, including a case definition?	YES	YES	YES	YES	YES	YES
3. Were the cases consecutive?	CD	CD	CD	YES	YES	NA
4. Were the subjects comparable?	YES	YES	YES	YES	YES	NA
5. Was the intervention clearly described?	YES	YES	YES	YES	YES	YES
6. Were the outcome measures clearly defined, valid, reliable, and implemented consistently across all study participants?	YES	YES	YES	YES	YES	YES
7. Was the length of follow-up adequate?	NA	NA	YES	NR	NA	YES
8. Were the statistical methods well-described?	YES	NO	YES	YES	YES	YES
9. Were the results well-described?	YES	NO	YES	YES	YES	YES
**TOTAL SCORE**	**FAIR**	**POOR**	**GOOD**	**GOOD**	**GOOD**	**FAIR**

Randomized Controlled Clinical Trial	Goldberg et al., 2003	Soran et al., 2010
1. Was the study described as randomized, a randomized trial, a randomized clinical trial, or an RCT?	YES	YES
2. Was the method of randomization adequate (i.e., use of randomly generated assignment)?	NR	YES
3. Was the treatment allocation concealed (so that assignments could not be predicted)?	NR	NR
4. Were study participants and providers blinded to treatment group assignment?	YES	YES
5. Were the people assessing the outcomes blinded to the participants' group assignments?	NO	NR
6. Were the groups similar at baseline on important characteristics that could affect outcomes (e.g., demographics, risk factors, co-morbid conditions)?	YES	YES

7. Was the overall drop-out rate from the study at endpoint 20% or lower of the number allocated to treatment?	NO	YES
8. Was the differential drop-out rate (between treatment groups) at endpoint 15 percentage points or lower?	NO	NR
9. Was there high adherence to the intervention protocols for each treatment group?	YES	YES
10. Were other interventions avoided or similar in the groups (e.g., similar background treatments)?	NR	YES
11. Were outcomes assessed using valid and reliable measures, implemented consistently across all study participants?	YES	YES
12. Did the authors report that the sample size was sufficiently large to be able to detect a difference in the main outcome between groups with at least 80% power	YES	YES
13. Were outcomes reported or subgroups analyzed prespecified (i.e., identified before analyses were conducted)?	YES	YES
14. Were all randomized participants analyzed in the group to which they were originally assigned, i.e., did they use an intention-to-treat analysis?	YES	YES
**TOTAL SCORE**	**POOR**	**POOR**

**Quasi-experimental study**	Celler et al., 2014	Jekel et al., 2016	Hayes et al., 2008	Rawtaer et al., 2020
1. Is it clear in the study what is the 'cause' and what is the 'effect' (i.e. there is no confusion about which variable comes first)?	YES	YES	YES	YES
2. Were the participants included in any comparisons similar?	YES	YES	YES	YES
3. Were the participants included in any comparisons receiving similar treatment/care, other than the exposure or intervention of interest?	YES	YES	YES	YES
4. Was there a control group?	YES	YES	YES	YES
5. Were there multiple measurements of the outcome both pre and post the intervention/exposure?	YES	YES	YES	YES
6. Was follow up complete and if not, were differences between groups in terms of their follow up adequately described and analyzed?	NR	YES	YES	YES
7. Were the outcomes of participants included in any comparisons measured in the same way?	YES	YES	YES	YES
8. Were outcomes measured in a reliable way?	YES	YES	YES	YES
9. Was appropriate statistical analysis used?	YES	YES	YES	YES
**TOTAL SCORE**	**GOOD**	**GOOD**	**GOOD**	**GOOD**

Abbreviations: CD, Cannot determine; N, No; NR, Not reported; Y, Yes.
