# Supplementary material for: Can Smart Home Technologies Help Older Adults Manage Their Chronic Condition? A Systematic Literature Review
Source: Int J Environ Res Public Health. 2023 Jan 10;20(2):1205. doi: 10.3390/ijerph20021205 (PMC9859495; doi:10.3390/ijerph20021205)
Supplement: Supplementary file 1 [file ijerph-20-01205-s001.zip › File S3.pdf]

Appendix 3: Description of IoT/AI/ML used in included studies

Author	IoT/AI/ML
Dawadi 2013	Eight different ML models, each of which learns a mapping between a single activity and its corresponding direct observation score. The score output of the algorithm was a sum of the eight individual activity scores generated by the eight different learning models
Fritz 2018	Combination of AI tools: Hidden Markov Models; Naive Bayes Classifier; Gaussian Mixture Models; Conditional Random Fields; Time-Series Forecasting Model; Decision Trees; Leave-one-home-out
Kuo 2012	-
Yu 2019	-
Chan 2004	-
Wakefield 2014	-
Alberdi 2018	**AI tools to behavior feature extraction:** Shannon entropy; gestalt pattern matching algorithm; Clinical Assessment using Activity Behavior algorithm; Principal Component Analysis; reliable Change Index computation.<br><br>**ML models for behaviour prediction**: SMOTEBoost; wrapper-based Rapidly Converging Gibbs sampler; Pairwise random algorithms; Principal Component Analysis
Urwyler 2017	-
Jekel 2016	-
Cavallo 2015	-
Celler 2014	-
Hayes 2008	-
Lazarou 2019	-
Goldberg 2003	-
Sacco 2012	-
Rawtaer 2020	-
Dahamen 2018	-
Soran 2010	-
Lazarou 2016	-

Abbreviations: IoT: Internet of Things; AI: Artificial Intelligence; ML: Machine Learning
